# Relationship of Neighborhood Deprivation and Outcomes of a Comprehensive ST‐Segment–Elevation Myocardial Infarction Protocol

**DOI:** 10.1161/JAHA.121.024540

**Published:** 2021-11-15

**Authors:** Chetan P. Huded, Jarrod E. Dalton, Anirudh Kumar, Nikolas I. Krieger, Nicholas Kassis, Michael Phelan, Kathleen Kravitz, Grant W. Reed, Amar Krishnaswamy, Samir R. Kapadia, Umesh Khot

**Affiliations:** ^1^ Department of Cardiology Saint Luke's Mid‐America Heart Institute Kansas City MO; ^2^ Department of Quantitative Health Sciences Lerner Research Institute Cleveland Clinic Cleveland OH; ^3^ Department of Cardiovascular Medicine Heart, Vascular, & Thoracic Institute Cleveland Clinic Cleveland OH; ^4^ Center for Healthcare Delivery Innovation Heart, Vascular, & Thoracic Institute Cleveland Clinic Cleveland OH; ^5^ Department of Emergency Medicine Emergency Services Institute Cleveland Clinic Cleveland OH

**Keywords:** disparities, door‐to‐balloon time, myocardial infarction, socioeconomic position, STEMI, Health Equity, Percutaneous Coronary Intervention, Revascularization

## Abstract

**Background:**

We evaluated whether a comprehensive ST‐segment–elevation myocardial infarction protocol (CSP) focusing on guideline‐directed medical therapy, transradial percutaneous coronary intervention, and rapid door‐to‐balloon time improves process and outcome metrics in patients with moderate or high socioeconomic deprivation.

**Methods and Results:**

A total of 1761 patients with ST‐segment–elevation myocardial infarction treated with percutaneous coronary intervention at a single hospital before (January 1, 2011–July 14, 2014) and after (July 15, 2014– July 15, 2019) CSP implementation were included in an observational cohort study. Neighborhood deprivation was assessed by the Area Deprivation Index and was categorized as low (≤50th percentile; 29.0%), moderate (51st –90th percentile; 40.8%), and high (>90th percentile; 30.2%). The primary process outcome was door‐to‐balloon time. Achievement of guideline‐recommend door‐to‐balloon time goals improved in all deprivation groups after CSP implementation (low, 67.8% before CSP versus 88.5% after CSP; moderate, 50.7% before CSP versus 77.6% after CSP; high, 65.5% before CSP versus 85.6% after CSP; all *P*<0.001). Median door‐to‐balloon time among emergency department/in‐hospital patients was significantly noninferior in higher versus lower deprivation groups after CSP (noninferiority limit=5 minutes; *P*_noninferiority_ high versus moderate = 0.002, high versus low <0.001, moderate versus low = 0.02). In‐hospital mortality, the primary clinical outcome, was significantly lower after CSP in patients with moderate/high deprivation in unadjusted (before CSP 7.0% versus after CSP 3.1%; odds ratio [OR], 0.42 [95% CI, 0.25–0.72]; *P*=0.002) and risk‐adjusted (OR, 0.42 [95% CI, 0.23–0.77]; *P*=0.005) models.

**Conclusions:**

A CSP was associated with improved ST‐segment–elevation myocardial infarction care across all deprivation groups and reduced mortality in those from moderate or high deprivation neighborhoods. Standardized initiatives to reduce care variability may mitigate social determinants of health in time‐sensitive conditions such as ST‐segment–elevation myocardial infarction.

**Nonstandard Abbreviations and Acronyms**
ADI: area deprivation index
CSP: comprehensive ST‐segment–elevation myocardial infarction protocol
D2BT: door‐to‐balloon time
GDMT: guideline‐directed medical therapy
SEP: socioeconomic position

**Clinical Perspective**

**What Is New?**

- A comprehensive ST‐segment–elevation myocardial infarction protocol was associated with improved guideline‐directed care for ST‐segment–elevation myocardial infarction across the spectrum of neighborhood deprivation levels.
- A comprehensive ST‐segment–elevation myocardial infarction protocol was associated with reduced in‐hospital ST‐segment–elevation myocardial infarction mortality driven by reduced mortality in patients from moderate or high deprivation neighborhoods.

**What Are the Clinical Implications?**

- Standardized protocols to reduce care variability may be associated with reduced health care disparities for high acuity, time‐sensitive conditions such as ST‐segment–elevation myocardial infarction.

Cardiovascular disease is the leading cause of mortality in the United States and worldwide, and acute ST‐segment–elevation myocardial infarction (STEMI) is a primary cause of death in patients with cardiovascular disease.^1^ It is well established that the care and outcomes of STEMI vary significantly based on a patient's socioeconomic position (SEP), even after adjusting for differences in baseline cardiovascular risk factors. Use of guideline‐directed medical therapy (GDMT), revascularization procedures, and achievement of guideline‐recommend door‐to‐balloon times (D2BTs) are significantly less common in patients with lower SEPs.^2^, ^3^, ^4^, ^5^, ^6^ These established disparities in care translate into increased rate of rehospitalization, worse quality of life, and higher short‐term and long‐term mortality after myocardial infarction in patients with lower SEPs.^2^, ^3^, ^4^, ^7^, ^8^, ^9^, ^10^, ^11^ Despite the established relationship between lower SEP and worse STEMI outcomes, there is no evidence that health care systems can mitigate these disparities or improve STEMI care and outcomes in lower SEP groups.

Given established relationships between social factors and health, there is an increasing recognition that strategies to prevent and treat cardiovascular disease must account for social determinants of health in addition to conventional biological risk factors.^12^ A recent scientific statement of the American Heart Association states that “the primary recommendation for future research is the design and evaluation of interventions, programs, and policies that address the social determinants of health…to shift the entire distribution of cardiovascular risk to lower levels and to target high‐risk individuals.”^13^

The Area Deprivation Index (ADI) of Kind and Buckingham^14^ is an established summary metric to effectively quantify neighborhood‐level SEP. The ADI comprises 17 data elements of education, employment, housing, and poverty obtained from US Census data and the American Community Survey data. Higher ADI scores indicate a higher level of deprivation, which corresponds with a lower SEP. A key strength of the ADI is that it can be geocoded to the Census block group level, which is a more granular geographic identifier than zip code and thus a better indicator of neighborhood‐level SEP. The purpose of this study was to evaluate the relationship between SEP, as measured using Census block group‐level ADI, and STEMI care and outcomes before and after implementation of a comprehensive STEMI protocol (CSP) within a regional STEMI system.

## Methods

### Study Population

Because of the sensitive nature of the data collected for this study, the data will not be made available to other researchers for the purpose of replicating the study results. This was an observational cohort study of consecutive patients with STEMI treated with primary percutaneous coronary intervention (PCI) at a single tertiary care medical center within a multihospital regional STEMI system between January 1, 2011, and July 15, 2019. Of 1847 consecutive patients with STEMI, 77 were excluded because of the inability to identify the place of residence, and 9 more were excluded with undocumented D2BTs, leaving a final study cohort of 1761 patients. The study size was selected to include 5 years of follow‐up data after CSP implementation. The multihospital regional STEMI system included 10 hospitals and 3 free‐standing emergency departments (EDs) within 60 miles by ground transport from the primary tertiary care hospital catheterization laboratory, where all PCI procedures were performed in this study. Baseline characteristics, procedural details, and in‐hospital outcomes were obtained from data entered prospectively by trained data abstractors into the American College of Cardiology National Cardiovascular Data Registry CathPCI database.^15^

### Comprehensive STEMI Protocol

On July 15, 2014, a CSP was implemented within the STEMI system to standardize 4 key aspects of STEMI care.^16^ First, before the CSP, the catheterization laboratory activation was authorized by cardiology providers after consultation with the ED physician. After CSP implementation, the ED physician was authorized to activate the catheterization laboratory for a diagnosis of STEMI without prior consultation with cardiology. Second, a STEMI Safe Handoff Checklist was introduced to provide clinical decision support for precatheterization laboratory care of STEMI patients. The checklist outlined high‐risk clinical alerts, key aspects of nursing care, and recommendations for GDMT administration. Third, a policy of immediate transfer to an immediately available catheterization laboratory was established, meaning that patients were not held in the ED awaiting catheterization laboratory readiness. Instead, the catheterization laboratory was expected to be immediately ready to accept a patient with STEMI at all times. Patients were immediately transferred to the catheterization laboratory once a diagnosis of STEMI was made. Finally, vascular access for cardiac catheterization in the context of STEMI was transitioned from an operator‐dependent approach to a “radial first” approach, meaning that operators were encouraged to use radial artery access when considered technically appropriate.^17^

### Assessment of Neighborhood Deprivation

**Figure 1:**
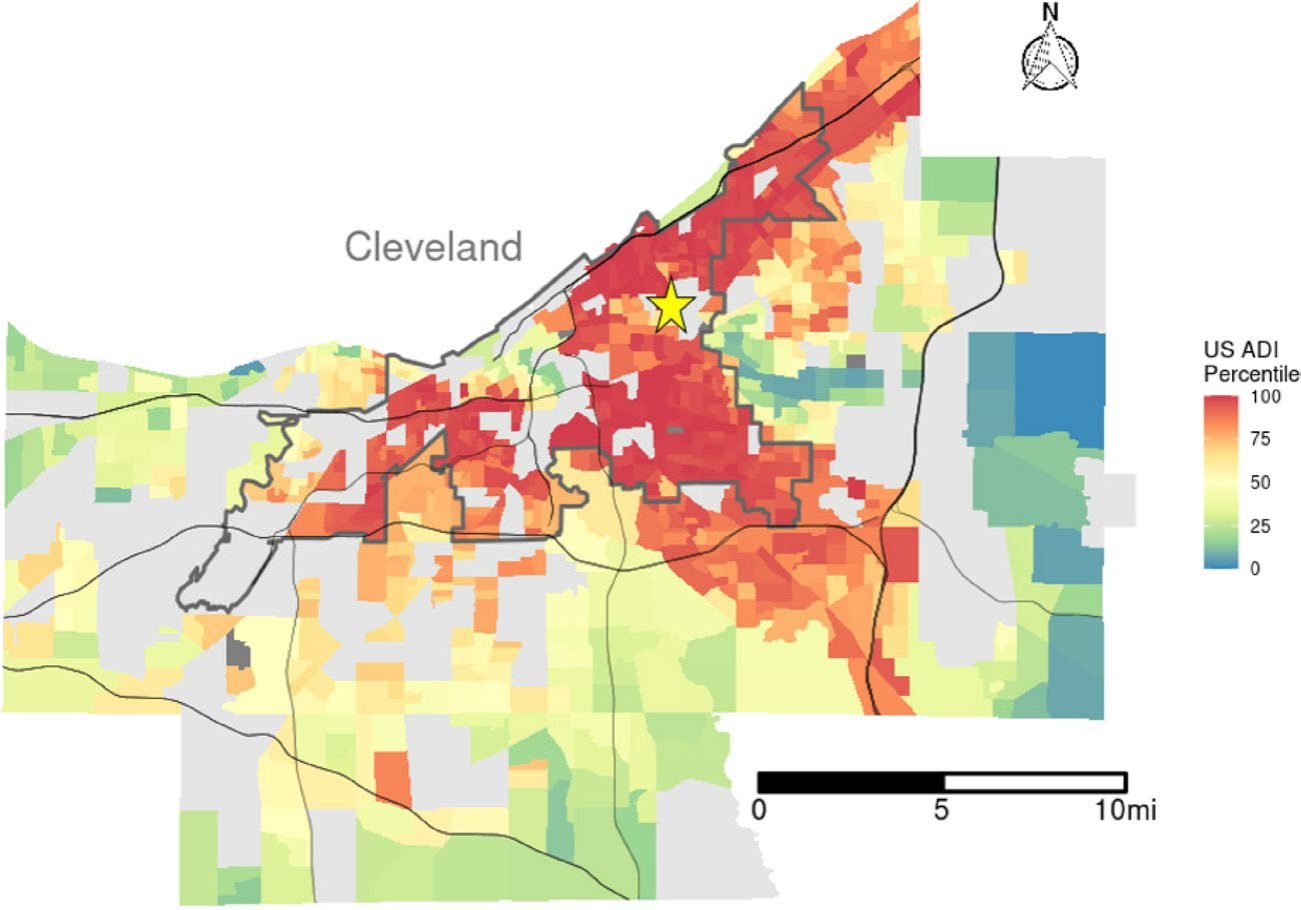
Geography of deprivation level and the ST‐segment–elevation myocardial infarction system. ADI of US Census blocks in the ST‐segment–elevation myocardial infarction system are shown based on the color gradient in the figure legend with red indicating higher ADI (higher deprivation level) and blue indicating lower ADI (lower deprivation level). Yellow star marks the location of the main campus catheterization laboratory where patients in this study were treated with percutaneous coronary intervention. Census tracts containing no patients with ST‐segment–elevation myocardial infarction in the study cohort are shown in gray. ADI indicates Area Deprivation Index; and US, United States.

Neighborhood deprivation was assessed for each patient at the US Census block group level based on geocoded addresses of residence using the ADI.^14^ For the purpose of this study, categories of low, moderate, and high deprivation were defined based on percentiles of the ADI distribution. To reflect the nonlinear impacts of severe poverty and disadvantage, low deprivation was defined as residence in a neighborhood with ADI ≤50th percentile among all US Census block groups, whereas moderate and high deprivation were similarly defined based on percentile ranges of 51% to 90% and >90%, respectively. The STEMI system evaluated in this study was inclusive of patients from across Northeast Ohio. Figure 1 shows US Census block groups in the region according to the level of neighborhood deprivation by ADI stratified by patient inclusion in this study. Figure S1 shows the US Census block group level ADI of the entire region without stratification by patient inclusion in the study.

### Outcomes

The primary process outcome of this study was D2BT, which was defined according to the CathPCI Registry standards as time of patient arrival at the ED until time of intracoronary device deployment that restored flow in the culprit vessel. For patients with in‐hospital STEMI, D2BT was the time from the first ECG showing STEMI to the time of first intracoronary device deployment restoring flow. The primary clinical outcome was in‐hospital mortality, which was recorded prospectively as part of data abstraction for the CathPCI Registry. Secondary process outcomes included treatment with GDMT before PCI and use of transradial access for PCI. GDMT before PCI was defined as administration of aspirin, a P2Y12 inhibitor (clopidogrel, prasugrel, or ticagrelor), and an anticoagulant (unfractionated or low‐molecular weight heparin) before arterial sheath insertion for PCI.

### Statistical Analysis

Baseline demographic and clinical characteristics were assessed separately across neighborhood deprivation groups, before and after implementation of the CSP, using standard univariable summary statistics. We similarly estimated univariable summary statistics on primary and secondary outcomes for these groups. Categorical variables are presented as number (percentage) and continuous variables with median (25th, 75th percentiles). This study was limited to in‐hospital follow‐up, and no patients were lost to follow‐up.

D2BT was assessed as an unadjusted continuous variable, and achievement of guideline‐directed D2BT goals (≤90 minutes for primary ED or in‐hospital presentation, ≤120 minutes for patients transferred from another ED or hospital) was evaluated as a binary categorical variable with comparisons of pre‐CSP versus post‐CSP groups by χ^2^ tests. Using quantile regression models, unadjusted D2BT was compared between deprivation groups with tests of noninferiority of a higher deprivation group relative to a lower deprivation group with a prespecified noninferiority margin of 5 minutes. Scatterplots of D2BT versus time were separately created by deprivation groups and STEMI presenting location (ED/in‐hospital versus transfer). Overlaid curves of D2BT median and quartiles as a function of time were derived from univariable interrupted time series quantile regression models in which slopes and intercepts were estimated separately by time period and presentation. Summary statistics of secondary outcomes are provided.

In‐hospital mortality before and after CSP implementation was compared in the overall population and among deprivation groups. To account for potential confounding attributed to changes in patient characteristics over time, risk‐adjusted in‐hospital mortality was evaluated using logistic regression models. The following candidate variables were selected for risk adjustment: age, sex, race, smoking, diabetes, prior myocardial infarction, prior heart failure, prior PCI, prior coronary artery bypass graft surgery, chronic obstructive pulmonary disease, chronic kidney disease, shock before PCI, and arrest before PCI. A model with all candidate variables was fit, and backward selection was performed to reduce the number of covariates. A total of 2 models for risk adjustment were constructed. Model 1 included the following patient demographics and comorbidities after backward selection: age, sex, race, smoking, diabetes, prior myocardial infarction, and prior heart failure. Model 2 included patient demographics and comorbidities from model 1 as well as early STEMI complications (shock before PCI, arrest before PCI) that may be modified by the STEMI protocol performance (ie, lower risk of shock or arrest before PCI as a result of a higher performing system with more prompt medical therapy and revascularization).^18^ Odds ratios (ORs), 95% CIs, and *P* values are provided.

The study was approved by the Cleveland Clinic Institutional Review Board, and written informed consent of study participants was not required. Dr’s Huded, Dalton, and Khot had full access to the data and take responsibility for its integrity and the data analysis.

## Results

Of 1761 patients included in the study, 512 (29.0%) resided in low‐deprivation neighborhoods; 718 (40.8%) resided in moderate‐deprivation neighborhoods, and 531 (30.2%) resided in high‐deprivation neighborhoods. Figure 1 shows the ADI distributions of the neighborhoods in the STEMI system and the location of the main campus catheterization laboratory, which is marked with a yellow star. The neighborhoods in close proximity to the main campus catheterization laboratory were predominantly high ADI (low SEP) neighborhoods, which corresponded to a higher rate of primary ED presentation and lower rate of transfer for PCI in the high‐deprivation group (Table 1).

**Table 1:** Baseline Demographic and Clinical Characteristics

Variable	Low deprivation		Moderate deprivation		High deprivation	
	Before CSP (n=174)	After CSP (n=338)	Before CSP (n=268)	After CSP (n=450)	Before CSP (n=232)	After CSP (n=299)
Presentation						
ED	20 (11)	45 (13)	64 (24)	75 (17)	78 (34)	134 (45)
In hospital	9 (5.2)	24 (7.1)	15 (5.6)	38 (8.4)	7 (3.0)	15 (5.0)
Transfer	145 (83)	269 (80)	189 (71)	337 (75)	147 (63)	150 (50)
Age, y	62.9 (55.4, 70.1)	62.7 (55.2, 70.0)	60.9 (52.1, 69.9)	61.3 (52.0, 70.6)	58.9 (50.7, 67.2)	61.0 (52.3, 69.7)
Male sex	125 (72)	242 (72)	185 (69)	304 (69)	147 (63)	179 (60)
Black race	6 (3.4)	7 (2.1)	43 (16)	80 (18)	148 (64)	180 (61)
Body mass index, kg/m^2^	28.0 (24.5, 31.3)	29.1 (25.2, 32.9)	28.3 (25.2, 32.5)	29.6 (26.0, 33.8)	28.7 (25.2, 32.4)	29.3 (25.1, 34.6)
Smoker	69 (40)	128 (38)	110 (41)	224 (51)	137 (59)	198 (67)
Diabetes	47 (27)	93 (28)	81 (30)	143 (32)	80 (34)	110 (37)
Prior MI	39 (22)	56 (17)	103 (38)	92 (21)	97 (42)	85 (29)
Prior heart failure	16 (9.2)	38 (11)	33 (12)	67 (15)	35 (15)	57 (19)
Prior PCI	22 (13)	72 (22)	58 (22)	107 (24)	46 (20)	75 (25)
Prior CABG	9 (5.2)	13 (3.9)	14 (5.2)	26 (5.9)	7 (3.0)	10 (3.4)
Peripheral artery disease	12 (6.9)	27 (8.1)	25 (9.3)	49 (11)	21 (9.1)	31 (10)
Cerebrovascular disease	18 (10)	37 (11)	27 (10)	47 (11)	38 (16)	42 (14)
COPD	14 (8.0)	29 (8.7)	25 (9.3)	63 (14)	34 (15)	49 (17)
Chronic kidney disease	35 (23)	76 (24)	65 (27)	106 (27)	56 (26)	52 (22)
Shock before PCI	23 (13)	24 (7.1)	33 (12)	29 (6.4)	30 (13)	20 (6.7)
Cardiac arrest before PCI	19 (11)	32 (9.5)	37 (14)	48 (11)	31 (13)	25 (8.4)

Categorical variables presented as number (percentage) and continuous variables as median (25th, 75th percentiles). CABG indicates coronary artery bypass graft surgery; COPD, chronic obstructive pulmonary disease; CSP, comprehensive ST‐segment–elevation myocardial infarction protocol; ED, emergency department; MI, myocardial infarction; and PCI, percutaneous coronary intervention.

The distribution of low‐deprivation, moderate‐deprivation, and high‐deprivation groups was similar between pre‐CSP (low, 174/674 [25.8%]; moderate, 268/674 [39.8%]; high, 232/674 [34.4%]) and post‐CSP (low, 338/1087 [31.1%]; moderate, 450/1087 [41.4%]; high, 299/1087 [27.5%]) time periods. In groups with higher neighborhood deprivation, we observed younger median age with a higher proportion of Black race (low deprivation, 2.5% [13/512]; moderate deprivation, 17.1% [123/718]; high deprivation, 61.8% [328/531]; *P*<0.0001) and female sex (low deprivation, 28.3% [145/512]; moderate deprivation, 31.9% [229/718]; high deprivation, 44.1% [234/531]; *P*<0.0001). The burden of cardiovascular risk factors and comorbidities such as smoking, diabetes, heart failure, prior myocardial infarction, peripheral artery disease, and cerebrovascular disease was greater with increasing levels of neighborhood deprivation despite younger age with higher deprivation status. When comparing pre‐CSP versus post‐CSP groups within deprivation strata, patient demographics and comorbidities were generally well balanced. However, pre‐CSP groups showed numerically higher proportions of prior myocardial infarction, cardiogenic shock before PCI, and cardiac arrest before PCI.

**Figure 2:**
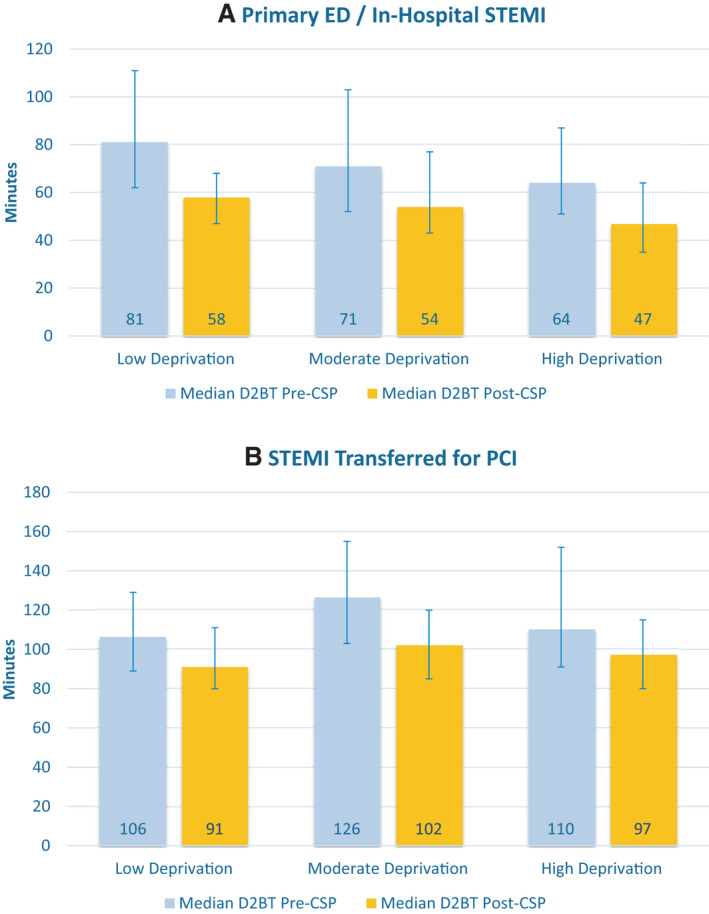
Median D2BTs by deprivation status and presenting location. **A**, D2BT among patients presenting to the primary ED or with in‐hospital STEMI before vs after comprehensive ST‐segment–elevation myocardial infarction protocol and stratified by deprivation status. **B**, D2BT among patients transferred for PCI before vs after comprehensive ST‐segment–elevation myocardial infarction protocol and stratified by deprivation status. D2BT indicates door‐to‐balloon time; ED, emergency department; PCI, percutaneous coronary intervention; and STEMI, ST‐segment–elevation myocardial infarction.

Figure 2 provides summary statistics of D2BT in patients pre‐CSP and post‐CSP implementation across levels of neighborhood deprivation. Median D2BTs were numerically lower after CSP implementation regardless of deprivation group or STEMI presenting location. In the pre‐CSP cohort, among patients presenting to the primary ED or with in‐hospital STEMI, D2BTs were numerically lower in groups with higher levels of deprivation. In the pre‐CSP cohort, among patients transferred from another hospital or ED for PCI, those with moderate deprivation showed the longest D2BT. The achievement of D2BT goals significantly increased in low‐deprivation (67.8% [118/174] before CSP versus 88.5% [299/338] after CSP; *P*<0.001), moderate‐deprivation (50.7% [136/268] before CSP versus 77.6% [349/450] after CSP; *P*<0.001), and high‐deprivation groups (65.5% [152/232] before CSP versus 85.6% [256/299] after CSP; *P*<0.001).

**Figure 3:**
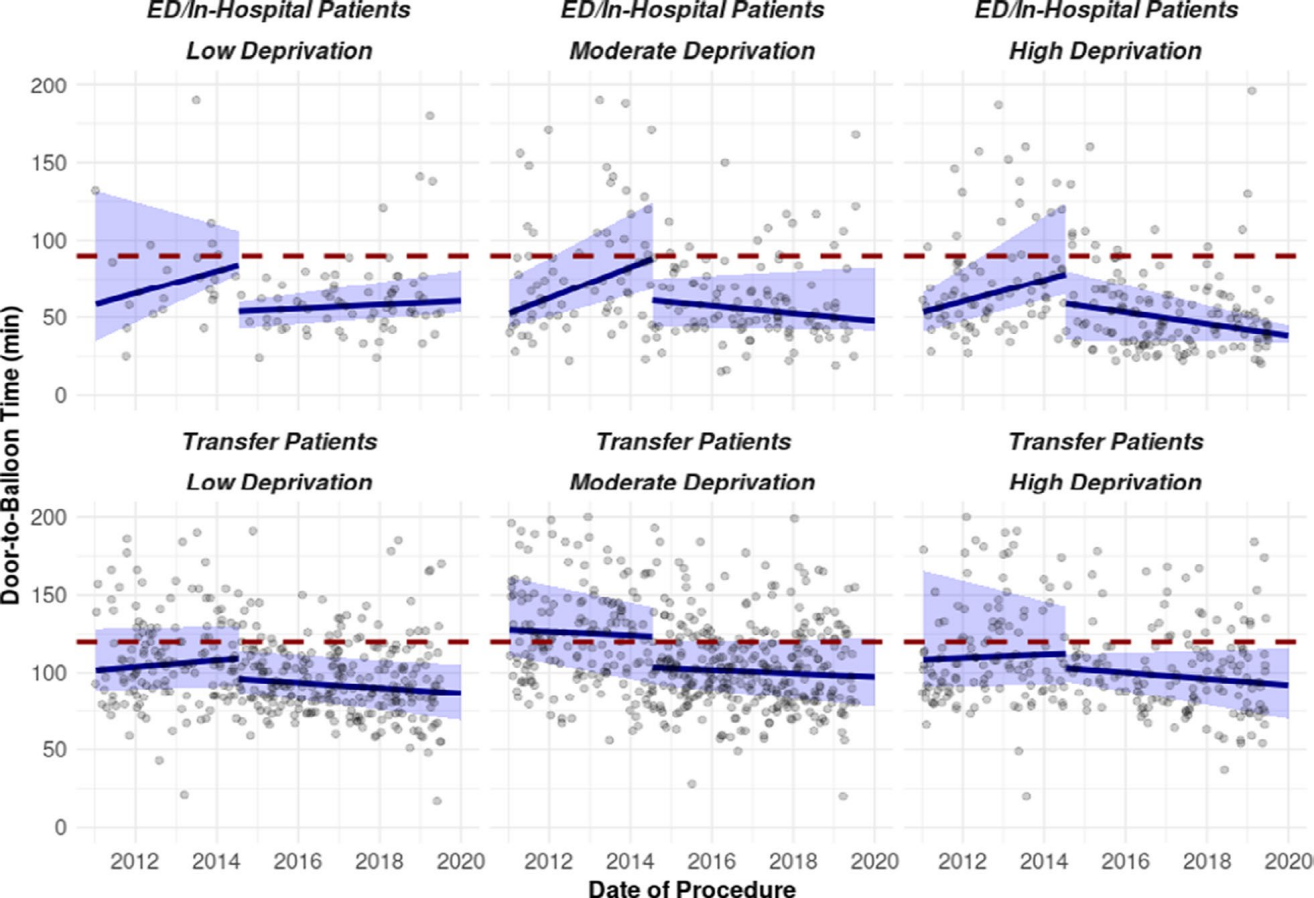
Door‐to‐balloon times by deprivation status during the study period. Scatterplots of door‐to‐balloon time vs time, separately provided by level of neighborhood deprivation and presentation (ED/in‐hospital vs transfer). Overlaid are curves of door‐to‐balloon time median and quartiles as a function of time as derived from univariable interrupted time series quantile regression models; for each quartile, slopes and intercepts were estimated separately by time period and presentation. ED indicates emergency department.

Figure 3 shows the distributions of D2BT with median and interquartile range over time separately provided by level of neighborhood deprivation and STEMI presentation type (primary ED/in‐hospital versus transfer). During the 5 years after CSP implementation, median D2BT performance was generally stable or improving in each group, and interquartile range was reduced regardless of deprivation level or STEMI presentation type.

The results of noninferiority testing of D2BT in higher versus lower deprivation groups are shown in Table 2. Among patients with primary ED/in‐hospital STEMI presentation, noninferiority of D2BT between high‐deprivation versus low‐deprivation groups was met before CSP implementation, but noninferiority was not satisfied in the comparisons of high versus moderate or moderate versus low deprivation. After CSP implementation, noninferiority of D2BT was met in each of the 3 pairwise comparisons of D2BT between high‐deprivation versus low‐deprivation groups. Among patients transferred for PCI, noninferiority of D2BT was observed in the comparison of high versus moderate deprivation, but not high versus low or moderate versus low deprivation comparisons, both before and after CSP implementation.

**Table 2:** Differences in Door‐to‐Balloon Time by Deprivation Status

Presentation	CSP	Comparison of deprivation groups	Difference in medians (min)	SE	*P* value* (noninferiority)
ED/in hospital	Before	High vs moderate	−7	6.9	0.12
		High vs low	−17	6.5	0.001
		Moderate vs low	−10	7.9	0.09
	After	High vs moderate	−7	3.7	0.002
		High vs low	−11	3.2	<0.001
		Moderate vs low	−4	3.7	0.02
Transfer	Before	High vs moderate	−16	4.7	<0.001
		High vs low	4	4.4	0.80
		Moderate vs low	20	3.9	>0.99
	After	High vs moderate	−5	2.9	<0.001
		High vs low	6	2.9	0.95
		Moderate vs low	11	2.6	>0.99

CSP indicates comprehensive ST‐segment–elevation myocardial infarction protocol; and ED, emergency department.

* *P* values reflect test of noninferiority of a specified higher deprivation group relative to the lower deprivation group (ie, *P*<0.05 reflects statistically significant noninferiority, as defined by a difference in median door‐to‐balloon time of not >5 minutes).

**Figure 4:**
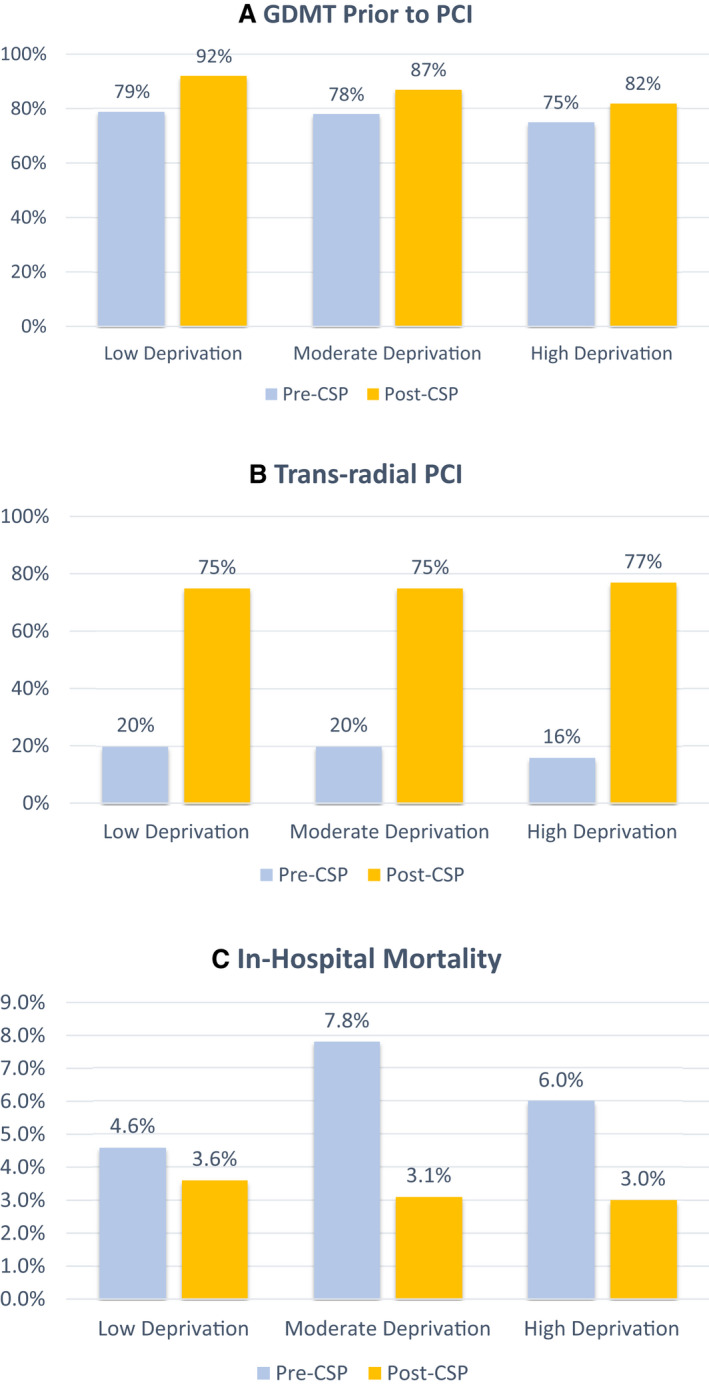
Additional clinical and process outcomes. **A**, Use of GDMT before PCI before and after CSP stratified by deprivation status. GDMT before PCI was defined as administration of aspirin, a P2Y12 inhibitor, and an anticoagulant medication before sheath insertion. **B**, Use of transradial PCI before and after CSP stratified by deprivation status. **C**, Unadjusted in‐hospital mortality before and after CSP stratified by deprivation status. CSP indicates comprehensive ST‐segment–elevation myocardial infarction protocol; GDMT, guideline‐directed medical therapy; and PCI, percutaneous coronary intervention.

Secondary process outcomes are shown in Figure 4. Use of GDMT before PCI increased in each deprivation group from before to after CSP, but the magnitude of increase was less in the higher deprivation groups. Adoption of transradial access for PCI was high post‐CSP implementation in each of the deprivation groups, with no meaningful observed differences in the magnitude of improvement.

Table 3 provides unadjusted and risk‐adjusted models of in‐hospital mortality after CSP implementation. We observed a significant in‐hospital mortality reduction after CSP implementation in the overall study cohort (before CSP, 6.4% [43/674] versus after CSP, 3.2% [35/1087]; unadjusted OR, 0.49 [95% CI, 0.31–0.77; *P*=0.002]; model 2 risk‐adjusted OR, 0.56 [95% CI, 0.33–0.93; *P*=0.03]), which was driven primarily by a significant mortality reduction in patients with moderate/high deprivation (7.0% [35/500] versus 3.1% [23/749]; unadjusted OR, 0.42 [95% CI, 0.25–0.72; *P*=0.002]; model 2 risk‐adjusted OR, 0.42 [0.23–0.77; *P*=0.005]), but not in those with low deprivation (4.6% [8/174] versus 3.6% [12/338]; unadjusted OR, 0.76 [95% CI, 0.31–1.90; *P*=0.56]; model 2 risk‐adjusted OR, 1.23 [95% CI, 0.44–3.42; *P*=0.70]). In‐hospital mortality was notably similar between deprivation groups after CSP implementation (low deprivation, 3.6% [12/338]; moderate deprivation, 3.1% [14/450]; high deprivation, 3.0% [9/299]).

**Table 3:** Risk‐Adjusted In‐Hospital Mortality After CSP Implementation

Comparison	Unadjusted, OR (95% CI)	Model 1: risk‐adjusted for demographic and comorbidities, OR (95% CI)	Model 2: risk‐adjusted for demographics, comorbidities, and shock/arrest before PCI, OR (95% CI)
All patients before vs after CSP	0.49 (0.31–0.77) (*P*=0.002)	0.41 (0.25–0.67) (*P*<0.001)	0.56 (0.33–0.93) (*P*=0.03)
Low deprivation before vs after CSP	0.76 (0.31–1.90) (*P*=0.56)	0.75 (0.29–1.94) (*P*=0.56)	1.23 (0.44–3.42) (*P*=0.70)
Moderate deprivation before vs after CSP	0.38 (0.19–0.76) (*P*=0.006)	0.31 (0.15–0.64) (*P*=0.002)	0.38 (0.17–0.83) (*P*=0.02)
High deprivation before vs after CSP	0.48 (0.21–1.14) (*P*=0.10)	0.37 (0.15–0.92) (*P*=0.03)	0.48 (0.18–1.27) (*P*=0.14)
Moderate/high deprivation before vs after CSP	0.42 (0.25–0.72) (*P*=0.002)	0.33 (0.19–0.59) (*P*<0.001)	0.42 (0.23–0.77) (*P*=0.005)

Model 1 included age, sex, race, smoking, diabetes, prior myocardial infarction, and prior heart failure. Model 2 included model 1 covariates+shock before PCI and arrest before PCI. CSP indicates comprehensive ST‐segment–elevation myocardial infarction protocol; OR, odds ratio; and PCI, percutaneous coronary intervention.

## Discussion

### Principal Findings

This observational cohort study evaluated changes in STEMI care and outcomes across levels of socioeconomic deprivation before and after implementation of a CSP within a regional STEMI system. Several principal findings were observed. First, increasing levels of socioeconomic deprivation, as measured with ADI geocoded to census block level, corresponded with a higher proportion of Black race, female sex, and cardiovascular comorbidities despite younger median age. Second, after CSP implementation, major improvements in D2BT performance were observed regardless of deprivation level. Among patients presenting to the primary ED or with in‐hospital STEMI, D2BT was noninferior between higher and lower deprivation groups after CSP implementation. Third, protocol‐directed improvements in the use of GDMT before PCI and transradial PCI were achieved in all deprivation groups, although the magnitude of GDMT improvements were blunted in those with higher deprivation. Finally, improvements in D2BT and other STEMI care metrics were associated with reduced in‐hospital mortality in patients from moderate or high deprivation neighborhoods.

### Increased STEMI Mortality in Higher Deprivation Groups

Multiple prior studies have shown higher mortality after acute myocardial infarction in those with lower SEP, but no prior study has evaluated an intervention to mitigate this disparity. Udell et al reported that lower census block level SEP was independently associated with higher in‐hospital mortality among patients with myocardial infarction in the Acute Coronary Treatment and Intervention Outcomes Network Registry—Get With The Guidelines program.^19^ Among those with STEMI specifically, Agarwal et al reported that lower zip‐code level SEP, as measured by median household income, was significantly associated with higher odds of in‐hospital mortality in the Nationwide Inpatient Sample.^10^ The association of lower SEP with higher mortality after myocardial infarction, both in the short term and long term, has been extensively confirmed.^2^, ^3^, ^4^, ^7^, ^9^, ^20^ Despite decades of literature establishing this relationship, Davis et al previously showed that “there are no studies describing attempts to improve acute coronary heart disease” as it relates to cardiovascular health disparities.^21^

### Importance of Reducing Care Variability in STEMI

We hypothesized that patients from higher deprivation neighborhoods may have worse D2BT performance during the pre‐CSP period and that post‐CSP improvements in D2BT performance among the higher deprivation groups would translate into lower mortality. However, we observed that the high‐deprivation group had the best D2BT performance before CSP. After CSP implementation, D2BTs were remarkably similar across deprivation groups, which was driven primarily by improved D2BT in patients with moderate or low deprivation. Despite this paradoxical relationship between deprivation status and D2BT, there was a significant reduction in mortality driven by mortality reduction in patients with moderate or high deprivation. These findings support the hypothesis that strategies that reduce care variability, independent of a relationship with D2BT, may be important to reduce STEMI mortality in vulnerable populations.

Although D2BT has historically been the focus of STEMI quality improvement initiatives, a singular focus on D2BT may exclude certain patients from realizing the benefits of such programs because D2BT metrics have traditionally excluded large groups of patients such as those transferred for PCI and those with nonsystem delays before PCI.^22^ The CSP in this study was applied to all patients regardless of clinical characteristics, presenting location, or D2BT reporting status. The multifaceted nature of the CSP (including interventions to improve GDMT and transradial PCI use) has been previously shown to have incremental prognostic value beyond the benefits achieved in D2BT alone.^23^

This study also confirmed that lower SEP was associated with higher proportions of female sex and Black race, highlighting that disparities in care and outcomes related to SEP are linked with other established health care disparities. This CSP has been previously associated with reductions in STEMI sex disparities as well, highlighting the potential benefit of a comprehensive and multifaceted approach to “leveling the playing field” with regard to STEMI disparities.^16^ Systems that standardize care may mitigate multiple health care disparities through a similar mechanism of reduced care variability and less influence of unconscious biases.

### Relationship Between Geography, ADI, and STEMI Performance

Neighborhoods in close proximity to the main campus catheterization laboratory in our STEMI system showed high ADI scores corresponding to lower SEP, which explains why a larger proportion of patients in the high‐deprivation group presented to the primary ED as opposed to being transferred for PCI. We hypothesize that a higher use of ED bypass with transportation directly to the catheterization laboratory via emergency medical services may explain the lower D2BTs in high‐deprivation patients during the pre‐CSP era as well as the blunted improvements in GDMT before PCI after CSP. More rapid D2BT and presentation directly to the catheterization laboratory in the high‐deprivation group likely contributed to less opportunity for checklist use in the ED and less time for up‐stream medication administration. Although ED bypass was encouraged among patients transported via emergency medical services, it should be noted that STEMI system improvements were applied to the entire regional health system and not solely to patients from high ADI neighborhoods close to the main campus.

### Strengths of the ADI as a Measure of SEP

One of the strengths of the present analysis is the use of ADI as a measure of SEP. Prior studies on this topic have used a variety of SEP metrics such as education level or predominantly household income. Although there is no established best method to measure SEP, there is an increasing recognition that multiple factors, rather than 1 individual factor, are associated with health in a complex and interrelated fashion.^13^, ^24^ There is a growing need to evaluate the relationship between multifactorial SEP metrics, such as ADI, with cardiovascular outcomes. We selected ADI for this study given that it is publicly available, multifactorial (incorporating 17 measures across areas of education, employment, housing quality, and poverty), and mappable to the US Census block group level.^14^ Although some prior studies have relied on zip‐code level SEP, mapping to the Census block group level allows for a far more specific characterization of neighborhood‐level SEP, which can vary widely even within a single zip code.

### Limitations

This study has several limitations. First, this study was conducted at a single center within a regional STEMI system, and the findings warrant validation in separate populations to confirm their generalizability. One of the challenges of generalizing these findings is the ability to provide 24/7 catheterization laboratory availability. Although our center is staffed with in‐house catheterization laboratory staff and fellows, other hospitals have used creative solutions to provide the necessary staff support using in‐house nurses, residents, or fellows to support the immediate transfer process.^25^, ^26^, ^27^ Second, the effect of the CSP was evaluated with a historical control, which may allow for confounding as a result of temporal changes unrelated to the study interventions. Risk adjustment was undertaken to reduce confounding related to changes in the population over time, but residual confounding is inherent to the observational nature of this work. Third, as discussed previously, we noted a paradoxical relationship between D2BT and deprivation status among patients presenting to the primary ED or with in‐hospital STEMI before CSP implementation. Although prior studies have reported that lower SEP is associated with longer reperfusion times such as D2BTs,^5^, ^6^, ^10^ we found that D2BTs were lower in those with higher deprivation (lower SEP) before CSP implementation, which may be related to more use of emergency medical services in those with higher deprivation. Fourth, this study made multiple comparisons of higher versus lower deprivation groups stratified into preintervention and postintervention periods, which increases the possibility of a type 1 error in rejecting a null hypothesis. Finally, clinical outcomes in this study were limited to in‐hospital follow‐up. Given the long‐term impact of social determinants of health, further work is necessary to confirm whether changes in STEMI process metrics and in‐hospital mortality translate into longer term benefits in those with lower SEP.

### Conclusions

A CSP was associated with improvements in D2BTs across all deprivation groups and reduced in‐hospital mortality in patients from moderate or high deprivation neighborhoods. Standardized quality improvement initiatives to reduce care variability may mitigate social determinants of health in time‐sensitive high‐acuity conditions such as STEMI.

## Sources of Funding

This project was funded by unrestricted philanthropic support to the Heart, Vascular, & Thoracic Institute Center for Healthcare Delivery Innovation and by the National Institute on Aging of the National Institutes of Health (R01AG055480). The content is solely the responsibility of the authors and does not necessarily represent the official views of the National Institutes of Health. The funding sources had no role in the design or conduct of the study; collection, management, analyses, or interpretation of the data; preparation, review, or approval of the manuscript; or decision to submit the manuscript for publication.

## Disclosures

None.

## Supporting information

Figure S1
