# Supplementary material for: Relationship of Neighborhood Deprivation and Outcomes of a Comprehensive ST‐Segment–Elevation Myocardial Infarction Protocol
Source: J Am Heart Assoc. 2021 Nov 15;10(24):e024540. doi: 10.1161/JAHA.121.024540 (PMC9075260; doi:10.1161/JAHA.121.024540)
Supplement: Supplementary file 1 — Figure S1 [file JAH3-10-e024540-s001.pdf]

SUPPLEMENTAL MATERIAL

Figure S1. Geographical Distribution of the Area Deprivation Index in the STEMI System.

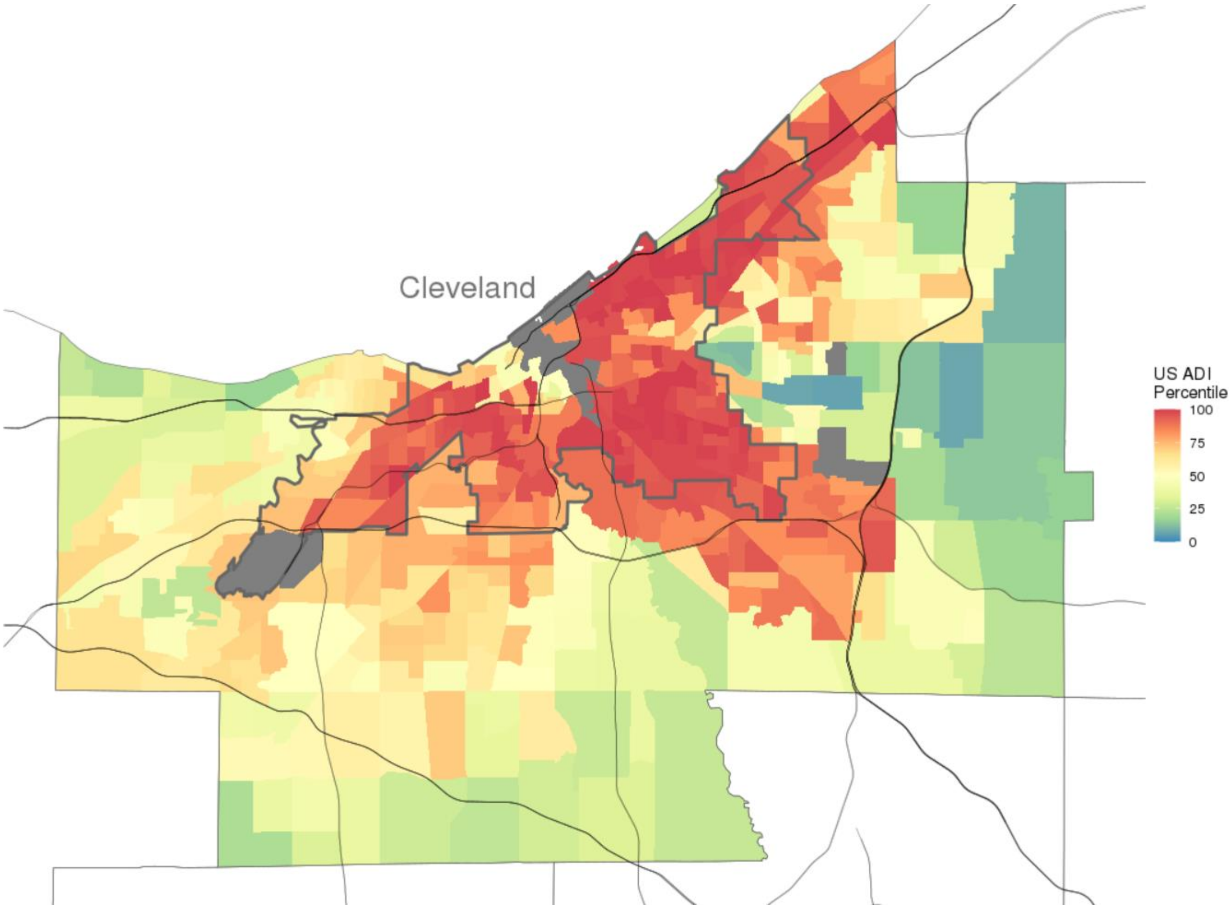

ADI of US Census blocks in the region are shown based on the color gradient in the figure legend with red indicating higher ADI (high deprivation level) and blue indicating lower ADI (lower deprivation level). ADI = area deprivation index. US = United States.
